# CO_2_ Laser Micromachining of PTFE-Based PCBs: Predictive Modeling of Kerf Depth Through Design of Experiments

**DOI:** 10.3390/mi17040404

**Published:** 2026-03-26

**Authors:** Giorgio Pellei, Paolo Di Stefano, Luca Mascalchi, Renzo Centi

**Affiliations:** 1 Department of Industrial and Computer Engineering and Economics, University of L’Aquila, 67100 L’Aquila, Italy; paolo.distefano@univaq.it; 2 Elco S.p.A., 67061 Carsoli, Italy; luca.mascalchi@elcopcb.com (L.M.); renzo.centi@elcopcb.com (R.C.)

**Keywords:** CO_2_ laser micromachining, PCB, PTFE, Design of Experiments, kerf depth, predictive model, process optimization

## Abstract

The escalating demand for miniaturization in electronics necessitates advanced laser micromachining for precise micro-via fabrication in PTFE-based PCBs. This study addresses challenges in controlling CO_2_ laser kerf depth in PTFE, a material known for properties that complicate material removal. Employing a two-level full factorial Design of Experiments, the effects of number of loops, aperture, and pulse duration were systematically investigated. This analysis revealed that while pulse duration statistically impacted ablation depth, the number of loops was operationally most critical due to its direct proportionality with kerf depth in PTFE, leveraging its low thermal conductivity. Aperture, defining the laser spot size, was often constrained by PCB geometric specifications. The predictive models developed demonstrated robust generalizability across different PTFE-based laminates. Validation of the production of PCBs achieved a 100% success rate in meeting geometric tolerances and surface integrity. This DoE-based framework establishes a process window, significantly reducing parameter identification time and scrap, thereby enhancing manufacturing yield.

## 1. Introduction

Laser micromachining has emerged as a critical enabling technology for the fabrication of high-precision components in advanced electronics manufacturing, particularly for the production of micro-vias and through-holes in printed circuit boards [1,2]. The continuous demand for miniaturization in telecommunications, micro-sensors, and biomedical devices has driven the development of increasingly sophisticated laser processing techniques capable of achieving micron-scale features with high repeatability and quality [3,4]. CO_2_ laser systems operating in the infrared spectral range (λ = 9.2–10.6 µm) have demonstrated significant advantages for processing polymer-based PCB materials due to their strong absorption by organic compounds and high processing efficiency [1,5].

PTFE-based PCB materials present particularly challenging processing characteristics due to their unique thermal and optical properties. PTFE exhibits high melting points and specific optical absorption characteristics that significantly influence laser–material interaction [6,7]. The complex nature of laser ablation mechanisms in polymers—encompassing both photochemical and photothermal processes—has been the subject of extensive research, with models attempting to describe these phenomena still evolving [8]. Recent comprehensive reviews have documented the diverse ablation behaviors across different polymer classes, emphasizing the necessity for material-specific modeling approaches [8].

The ability to accurately predict and control kerf depth during CO_2_ laser processing of PTFE-based materials is crucial for achieving reliable micromachining operations and ensuring process yield. Previous investigations have demonstrated that pulsed-laser ablation rates of polymers are dominated by optical penetration absorption during initial periods and follow Beer’s law relationships with respect to laser fluence [9]. Analytical models based on heat diffusion equations have been developed to estimate melting depths and thermal effects during laser processing, accounting for material-specific thermal properties such as optical kerf depth and thermal diffusivity [10]. Recent numerical simulation studies have further advanced our understanding of heat-affected zone formation and material removal mechanisms that are conceptually relevant to polymer processing [11].

Design of Experiments methodologies have proven to be powerful tools for optimizing complex laser manufacturing processes, offering substantial advantages over traditional “change one separate factor at a time” approaches [12,13]. This approach enables efficient exploration of parameter spaces, identification of influential factors, quantification of interaction effects, and development of predictive models with a minimum number of experimental trials, thereby reducing time, cost, and resource requirements compared to exhaustive testing [14,15].

Recent applications of DoE to laser processing have demonstrated its effectiveness across diverse materials and processes. Studies employing Box–Behnken designs have optimized CO_2_ laser drilling parameters such as power and exposure time to achieve desired depth, entry diameter, and heat-affected zone characteristics in polycarbonate samples [5]. Numerical simulations coupled with statistical analysis have been utilized to investigate the influence of laser power, cutting speed, and focal plane position on kerf geometry and thermal effects in thermoplastic materials [16]. Artificial neural networks have also been employed alongside statistical methods for predicting laser-machined micro-channel dimensions, with comparative studies highlighting the complementary strengths of machine learning and traditional DoE approaches [17].

The manufacturing landscape continues to evolve, with process optimization extending into multilayer PCB fabrication and advanced material systems [18]. Recent work has demonstrated the successful application of statistical DoE methodologies to multilayer PCB fabrication processes [18]. The present study focuses on the development of predictive mathematical models for CO_2_ laser kerf depth in PTFE-based PCB materials through a comprehensive DoE approach [19]. Specifically, a two-level full factorial design was implemented to systematically examine the influence of aperture, number of loops, and pulse duration on the resulting kerf depth, enabling the quantification of both main effects and parameter interactions within the experimental domain [20]. This systematic approach facilitates the construction of a regression model that correlates these process variables with the observed kerf depth, providing a quantitative framework for process optimization and quality control in PTFE-based PCB manufacturing [21].

This experimental activity was carried out in collaboration with Elco S.p.A., a PCB production factory based in Italy, and, due to industrial secrecy, the exact equations of the models cannot be disclosed, but their generalized form—which incorporates linear terms for the input parameters—can be presented. However, CO_2_ laser routing represents a critical micromachining step: insufficient selectivity can result in incomplete routing and potential PCB defects, while excessive energy causes heat-related phenomena that yield non-compliant PCBs. Despite its industrial relevance, quantitative process models for CO_2_ laser-based contouring of PTFE-based PCBs remain limited. To address this, a DoE-based predictive framework was proposed to define a process window for laser micromachining of PTFE-substrate PCBs, enabling controlled material removal. Although Design of Experiments is widely used for modeling laser–material interactions, which also consistently shows that parameters such as power, speed, and the number of passes are the primary drivers of kerf depth [5,16,22], the current literature predominantly focuses on micro-drilling or single-pass cutting of homogeneous polymers such as PMMA, PC, and PE [5,23,24]. A significant research gap persists regarding multi-pass routing of specialized high-frequency substrates like PTFE-based PCBs. These materials pose unique challenges due to their low surface energy, high thermal stability, and the presence of ceramic or fiberglass reinforcements, which significantly alter the ablation threshold and energy accumulation dynamics compared to standard plastics [7,25]. Unlike previous empirical studies that prioritize kerf width or surface roughness in single-pass operations [26,27], this work explicitly investigates the influence of parameters such as the number of loops, pulse duration, and aperture to predict cumulative kerf depth. By comparing models across different PTFE grades, this study establishes a quantitative methodology for defining industrial process windows in PTFE-based PCB manufacturing—a capability that is currently missing from the existing empirical and theoretical literature [1,17]. The predictive model is used as a tool to identify a stable micromachining window rather than as a transferable numerical recipe. It is not intended as a universal physical model but should primarily be considered an empirical tool valid within a defined parameter window, which represents the most commonly used setting on the laser system employed in this experiment, one of the most widely used laser systems in PCB manufacturing factories.

Therefore, the discussion focuses on process trends, parameter interactions, and robustness.

Thanks to this experimental activity, it was possible to substantially reduce the time required to identify optimal parameters for micromachining of this PCB type while minimizing the risk of scrap generation due to poor parameter choices.

## 2. Materials and Methods

The experimental approach employed a Design of Experiments framework to systematically investigate the influence of key process parameters on the kerf depth achieved during CO_2_ laser contouring of PTFE-based PCBs. The selection of aperture, number of loops, and pulse duration as the primary control factors was informed by preliminary screening tests, which identified these parameters as having the most significant impact on material removal rates and feature quality in PTFE substrates: aperture determines the laser spot size on the working board, the number of loops controls the cumulative energy exposure, and pulse duration governs the duration of each laser pulse, all of which are critical for managing thermal input and material removal efficiency. Parameter ranges were determined from preliminary trials to ensure effective material ablation without substrate damage from excessive energy deposition. In particular, the maximum number of passes was limited to 20 to prevent excessive charring of the PTFE ceramic filler, while the minimum was set to achieve a kerf depth compatible with the typical minimum thickness of PTFE laminates. The pulse duration was set in the range of 5–9 μs to balance material removal efficiency with thermal management: shorter durations minimize heat diffusion, while longer ones increase the risk of thermal damage to the PTFE substrate. To better describe the aperture parameter, the CO_2_ laser system used in this study features a specialized spatial-filtering mechanism for controlling the incident beam diameter. This mechanism consists of a rotating drum containing brass reductors (apertures) of varying diameters. By selecting a specific reductor, the system physically clips the raw laser beam, thereby controlling the effective beam spot size delivered to the focusing optics. This mechanical adjustment is essential for modulating the beam diameter incident on the workpiece. Accordingly, the full aperture range of 127–254 μm was selected to evaluate the effect of varying laser spot size on ablation efficiency.

**Figure 1:**
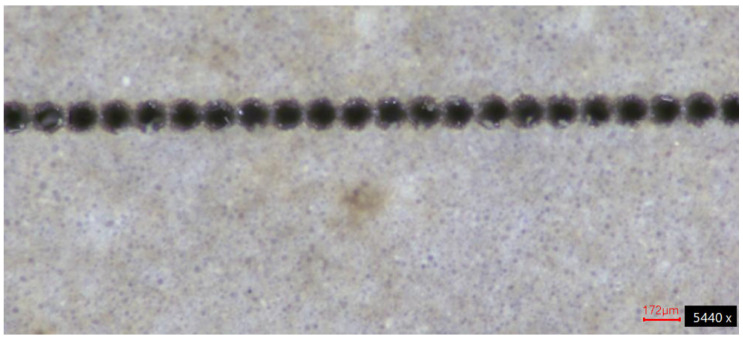
Low spot-overlap ratio related to excessive scanning speed values.

For the smaller and larger apertures, the power was fixed at 42.32 W and 54.90 W on average, respectively. The frequency was fixed at 3.3 kHz. Lastly, the scanning speed was fixed at 400 mm/s, as lower values cause unnecessary thermal energy accumulation that can degrade the PTFE substrate, whereas higher values compromise contouring precision, producing a low spot-overlap ratio where discrete ablation points fail to merge into a continuous contour, as shown in Figure 1 for a scanning speed of 500 mm/s, aperture of 127 μm, pulse duration of 9 μs, and 10 loops.

For this reason, scanning speed was excluded from the experimental variables, since the value of 400 mm/s represented the equipment manufacturer’s designated ‘sweet spot’ for optimized industrial contouring. This ensured a constant spot-overlap ratio throughout the trials and isolated the primary influences of the studied parameters on the kerf depth.

### 2.1. Materials

- Laser cutting machine:

The Laser Combi Drill 350 by Schmoll Maschinen (Rödermark, Germany) was utilized, equipped with dual laser sources: a UV laser for copper removal and hole cleaning and a CO_2_ laser for dielectric removal.

For the CO_2_ laser source, a Coherent Diamond J-5V-V (Coherent Deutschland GmbH, Dieburg, Germany) was used. The laser wavelength was 9.4 μm. The beam ellipticity ratio was $0.83\leq \frac{w_{x}}{w_{y}}\leq 1.2$. The full-angle beam divergence was less than 2.2 mrad for each aperture. The spatial mode was TEM_00_ (M < 1.2). The maximum duty cycle was 9.1%. The laser beam waist diameter of 7.0 ± 1.0 mm (at 1/e^2^) was introduced to the PCB substrate using a galvanometric scanner with an F-theta lens, achieving a variable spot size, depending on the selected aperture.

- Laminates:

A double-sided Rogers RO3003 laminate (Rogers Corporation, Chandler, Arizona, AZ, USA) with 1.573 mm dielectric thickness, where both top and bottom copper clads had been removed by etching, was selected to evaluate the interaction between the CO_2_ laser and PTFE materials. Rogers RT/Duroid 5880 (Rogers Corporation, Chandler, Arizona, AZ, USA)and RT/Duroid 6002 laminates (Rogers Corporation, Chandler, Arizona, AZ, USA) (both with 1.573 mm thickness) were further employed to assess the model’s robustness.

- Test coupon:

A 20 × 20 mm test coupon was obtained from the panel, consisting of a grid with 10 individual cuts, each corresponding to a specific DoE condition. In order to capture the variability inherently associated with both the material and the manufacturing process—such as differences in laminate composition and fluctuations in the laser beam—and thus improve the robustness and applicability of the predictive model to industrial production, the coupon grids were distributed over the panel surface, and the specimens were extracted from randomly selected positions.

The software, optical microscope, grinding machines, and resins are identical to those described in another of our works [28].

### 2.2. Methods

- Design of Experiment (DoE):

To systematically assess the effects of key CO_2_ laser parameters on kerf depth in PTFE laminate, a Design of Experiments method using two-level full factorial designs was implemented. This approach enabled comprehensive analysis of the individual and interactive effects of these parameters (specifically, the number of loops, aperture, and pulse duration) on cut kerf depth in the PTFE-based substrates. Only parameters with p<0.05 were included in the final model.

**Figure 2:**
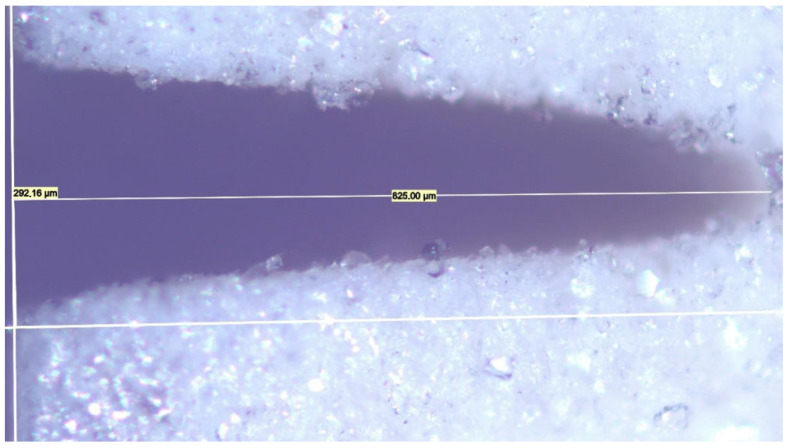
Example of CO_2_ laser kerf depth (825.00 μm) and width (292.16 μm) measurements corresponding to a DoE trial conducted with a 254 μm aperture, 9 μs pulse duration, and 20 loops.

- Measurement approach of the cross-sections:

For each experimental condition defined in the DoE matrix, five separate cuts were performed and measured to ensure statistical significance. These five data points represent repeated measurements from a single processing run, specifically used to estimate the short-term repeatability of the laser–material interaction and the precision of the metallographic sectioning and optical measurement techniques. For each DoE condition, the standard deviation of the repeated measurements was calculated; the maximum value observed across all conditions was 1.5 μm, confirming that experimental noise and measurement uncertainty are negligible compared to the parametric effects identified by the model. A predictive model for CO_2_ laser kerf depth ($h_{p}$) in PTFE substrates was then developed, using the arithmetic mean of these five measurements as the response output for ANOVA and regression modeling (Figure 2).

## 3. Results and Discussion

In this section, the results derived from the DoE are presented, focusing on the statistical significance of the investigated parameters and their implications for predicting the CO_2_ laser kerf depth across PTFE-based laminates. Specifically, this analysis contributes to the formulation of predictive mathematical functions that correlate operational parameters—such as the number of loops, pulse duration, and aperture—with the resulting ablation depth in the studied substrates.

### 3.1. Rogers RO3003 Substrate

The experimental conditions of the 2^3^ full factorial DoE are presented in Table 1.

As described previously, for each DoE condition, five cuts were measured to ensure statistically significant results, and their means were used as the DoE response outputs, as shown in the following Table 2.

**Figure 3:**
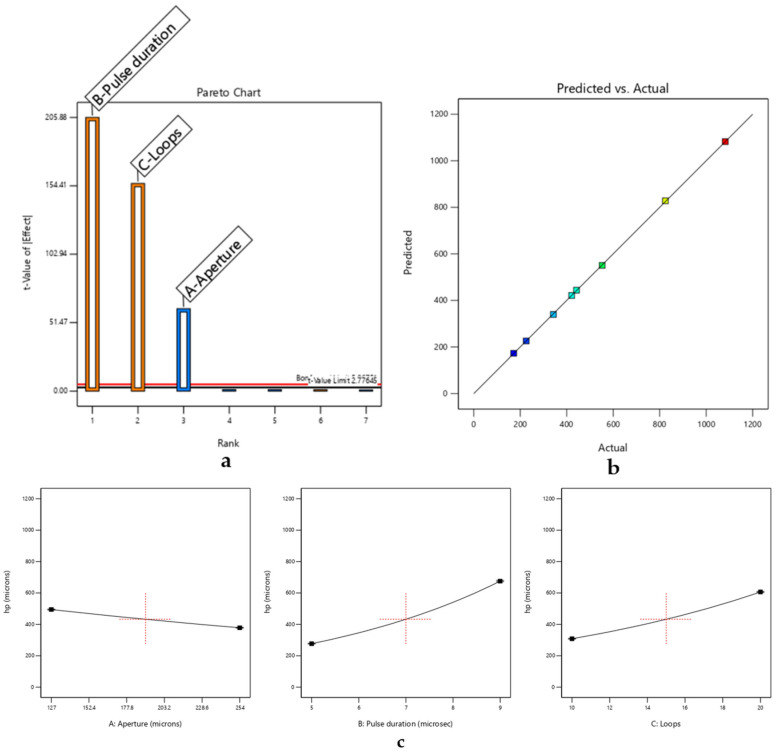
DoE-based analysis of CO_2_ laser cutting of RO3003, highlighting (**a**) dominant parameters affecting removal selectivity and (**b**,**c**) process trends that delineate the stable micromachining window.

ANOVA was performed to assess the significance of the model terms, yielding the results detailed in Table 3 and Figure 3.

Figure 3a presents the Pareto chart of standardized effects, highlighting the relative importance of the investigated process parameters. Pulse duration and the number of loops show the strongest influence on kerf depth, exceeding the statistical significance threshold, while aperture exhibits a lower but still relevant contribution. These aspects are examined in greater detail below.

Figure 3b compares predicted and experimental values of kerf depth. The close alignment of the data points along the diagonal confirms the good agreement between the model and experimental observations, indicating a reliable predictive capability within the investigated parameter range.

Figure 3c illustrates the main effects plot, further elucidating the individual impact of each parameter on the response variable.

The obtained model for $h_{p}$ can be expressed as a base-10 logarithmic law of the form ${log}_{10}(h_{p})$, where the statistically significant factors are as follows:

- Pulse duration, with a positive effect on the response;
- Loops, with a positive effect on the response;
- Aperture, with a negative effect on the response.

According to the ANOVA results, the second-order (2-way) and third-order (3-way) interactions—representing the synergistic effects between combined process factors—were not statistically significant for predicting kerf depth (*p* > 0.05). Consequently, these terms were excluded from the predictive model.

The generalized equation of the model is as follows:

(1) $${log}_{10}\left(h_{p}\right)=\alpha_{0}+\alpha_{1}\;*\;B+\alpha_{2}\;*\;C-\alpha_{3}\;*\;A$$

It is significant to highlight that the measured kerf depths exhibited a near-linear scaling with respect to the number of loops, where increments in loop count resulted in approximately proportional increases in depth.

While it may appear intuitive that, for example, doubling the loops results in a doubling of the kerf depth, such a direct near-linear relationship is notably rare in multi-pass laser micromachining [19,29]. In most polymeric and composite systems, the ablation process typically enters a regime of depth saturation, where the effectiveness of each successive pass decreases as the aspect ratio increases, often due to plume shielding and the physical difficulty of debris ejection from deep channels [19,30]. Alternatively, many materials exhibit a thermal incubation effect, where cumulative energy deposition leads to an exponential or non-linear increase in the ablation rate over successive pulses [31,32].

**Figure 4:**
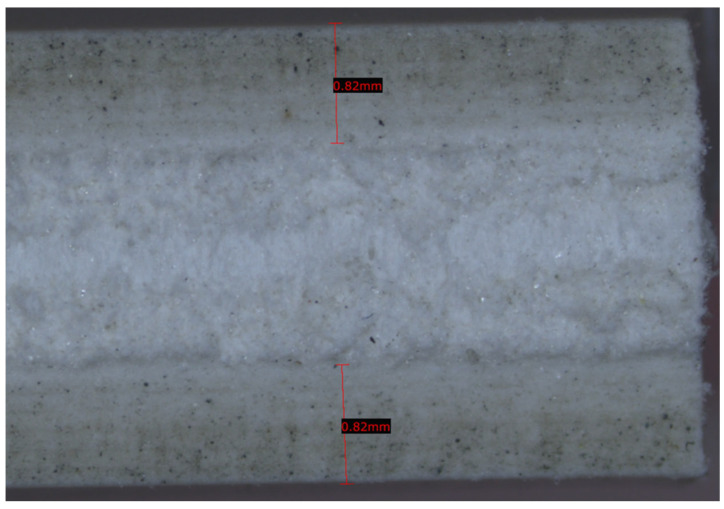
Cross-sectional view of a CO_2_ laser-machined kerf in PTFE-based material, obtained by mechanically opening the sample. The laser-affected regions at the top and bottom surfaces (≈0.82 mm) are compared with the untreated core. The absence of significant color variation indicates limited thermal degradation and suggests a negligible heat-affected zone (HAZ) within the investigated process window defined by the DoE.

The near-linearity observed in this study suggests that for the investigated PTFE-based substrates, heat-associated interference and shielding effects are negligible [7,9]. This is evident in Figure 4, where the wall of the opened kerf exhibits only a very faint darker color compared to the unprocessed PTFE substrate, confirming negligible heat-associated effects.

This deterministic behavior represents a major industrial advantage; it enhances the repeatability and reliability of the routing operation, allowing for highly precise kerf depth control.

This behavior is attributable to PTFE’s inherently low thermal conductivity [33], which limits heat diffusion, combined with optimized process parameters—such as short pulse durations (5–9 μs) and a fixed scanning speed of 400 mm/s—that prevent thermal accumulation and ensure independent energy delivery per loop. In contrast, aperture and pulse duration exhibited non-linear relationships with kerf depth. Increasing the laser spot size or pulse duration did not yield proportional gains in material removal, due to energy density distribution effects and thermal saturation thresholds within the PTFE substrate.

Upon analyzing the effects of the CO_2_ laser parameters on kerf depth in PTFE-based PCBs, it is crucial to distinguish between a parameter’s statistical effect and its operational criticality in production. While pulse duration can exhibit the most significant effect on the resultant ablation depth (60.02% of contribution to the total variance in kerf depth), the number of loops is often considered the most critical factor from a production standpoint. This is primarily because the number of loops directly governs the kerf depth, exhibiting a near-linear proportionality, as discussed before. This linearity allows for precise control over the target depth, making it indispensable for avoiding insufficient ablation or thermal damage that can occur with excessive energy delivery.

Pulse duration, in turn, also influences the ablation depth per pulse, as longer durations can increase material removal. This characteristic allows pulse duration to be leveraged for process time optimization, as a greater ablation volume per pulse can reduce the overall number of loops required to achieve a specific kerf depth. However, its influence can be non-linear and governed by thermal saturation limits. The aperture, which defines the laser spot size and thus the energy density on the material, also significantly impacts the ablation process. Nevertheless, its adjustability in production is often constrained by the geometric features and design specifications of the PCB itself, as the minimal dimensions of resulting structures are closely related to the laser beam size. Therefore, while all three parameters influence the final outcome, the number of loops provides the most direct and operationally critical control for achieving precise kerf depths in the CO_2_ laser micromachining of PTFE-based PCBs.

Residual diagnostics were performed to assess the validity of the regression assumptions and confirm the reliability of the ANOVA results. The analysis is reported in the Supplementary Materials (Figures S1–S5). The logarithmic transformation of the response variable was adopted based on the Box–Cox analysis to improve residual normality and variance stability (Figure S1). The normal probability plot showed an approximately linear distribution of residuals (Figure S2), while the residuals vs. predicted plot indicated an overall stable variance pattern (Figure S3). A single observation exhibited a relatively large studentized residual, which is attributed to the very small residual variance of the model, where minor experimental deviations can produce amplified standardized residual values. Nevertheless, the residuals vs. run plot did not reveal any systematic structure, confirming the independence of observations (Figure S4). Finally, Cook’s distance analysis did not identify influential observations (Figure S5), supporting the robustness of the fitted model.

### 3.2. Model Validation on Rogers RT/Duroid 5880

To assess the generalizability of the predictive model developed for the RO3003 substrate, validation experiments were conducted on the Rogers RT/Duroid 5880 material using the same parameter settings defined by the factorial design. The experimental results obtained for RT/Duroid 5880 were compared against the predicted values derived from the RO3003 model to evaluate prediction accuracy across different PTFE-based laminates.

Then, the same DoE was replicated for this material, except that the maximum number of loops was reduced to 15 (Table 4), as the most aggressive condition in the original design would have exceeded the typical maximum thickness of PTFE-based laminates processed at Elco S.p.A.

It should be noted that while the maximum number of loops for the RT-5880 validation was slightly reduced compared to the initial RO3003 trials, all validation points remained strictly within the calibrated design space (10–20 loops). Because the model is based on interpolation within this defined parameter window, the reduction does not compromise direct comparability; rather, it demonstrates the model’s ability to maintain high predictive accuracy at any point along the identified regression curve for different PTFE-based substrates.

Again, for each DoE condition, five cuts were measured to ensure statistically significant results, and their means were used as the DoE response outputs, as shown in the following Table 5.

Lastly, ANOVA was performed to assess the significance of the model terms, yielding the results detailed in Table 6 and Figure 4.

**Figure 5:**
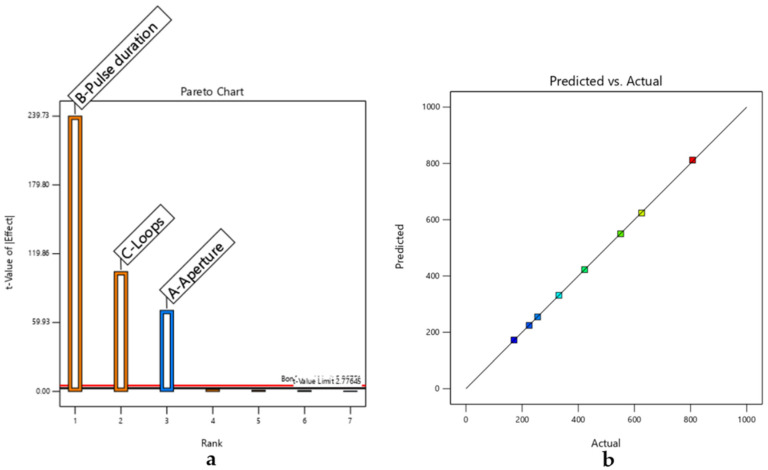

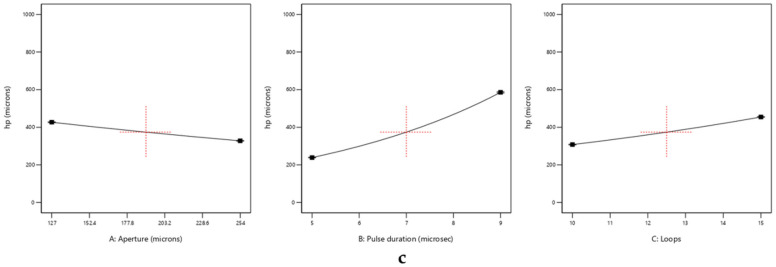
DoE-based analysis of CO_2_ laser cutting of RT/Duroid 5880, highlighting (**a**) dominant parameters affecting removal selectivity and (**b**,**c**) process trends that delineate the stable micromachining window.

The observations concerning Figure 5 align with the principal findings from the initial RO3003 analysis, thereby underscoring the robustness of the parameter–kerf depth relationships across comparable PTFE-based substrates.

The obtained model for $h_{p}$ in RT/Duroid 5880 can also be expressed as a base-10 logarithmic law of the form ${log}_{10}(h_{p})$, where the statistically significant factors are as follows:

- Pulse duration, with a positive effect on the response;
- Loops, with a positive effect on the response;
- Aperture, with a negative effect on the response.

Again, the second-order (2-way) and third-order (3-way) interactions were found not statistically significant for predicting kerf depth (*p* > 0.05), and consequently, these terms were excluded from the predictive model.

Interestingly, this model is essentially superimposable on the RO3003 model, with the same significant factors identified and nearly identical equation coefficients, suggesting that the underlying ablation mechanisms are consistent across these PTFE-based laminates despite their different material compositions. Clearly, the same physical and process considerations (as well as the generalized form of the equation) developed for the RO3003 model also apply to the RT/Duroid 5880 model.

Residual diagnostics were also performed for the validation model to verify the underlying regression assumptions and confirm the reliability of the statistical analysis. The corresponding plots are reported in the Supplementary Materials (Figures S6–S10). The diagnostic analysis confirmed the adequacy of the model: the normal probability plot showed a satisfactory linear alignment of residuals (Figure S6), the residuals vs. predicted values indicated constant variance across the response range (Figure S7), and the Box–Cox analysis supported variance stability (Figure S8). Furthermore, the residuals vs. run plot did not reveal any systematic pattern, confirming the independence of observations (Figure S9). Cook’s distance analysis did not highlight influential observations (Figure S10), further supporting the robustness of the fitted model.

This consistency validates the robustness of the predictive equations and confirms the absence of overfitting.

### 3.3. Model Validation of PCB Production 

To further evaluate the predictive capability of the developed models under real-world manufacturing conditions, validation tests were performed on production printed circuit boards made from a third PTFE-based substrate. A final quality assurance check verified that the laser ablation process met the required specifications for dimensional accuracy and surface integrity. The validation procedure involved comparing the measured kerf depths on production panels with the values predicted by the models, evaluating whether the target kerf depth was achieved within acceptable tolerance limits while maintaining the structural integrity of the substrate features. To achieve the target kerf depth, a parameter set was selected to produce a kerf 40 μm deeper than the PCB thickness. To assess whether the CO_2_ laser, according to the model, actually penetrated 40 μm more, a backup PTFE-based panel (RO3003) was placed beneath the workpiece, and the excess kerf depth was then measured. The parameters obtained from the model were as follows:

− Loops: 13;
− Pulse duration: 9 μs;
− Aperture: 127 μm.

Using these parameters, the model predicted a kerf depth of 675 μm.

**Figure 6:**
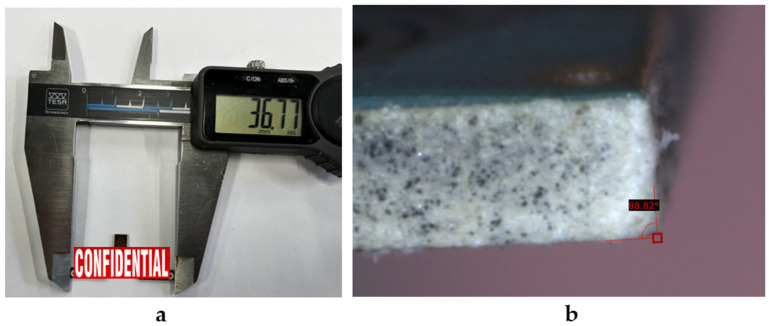
PCB dimensions (**a**) and kerf angle measurement (**b**) of a PCB sample.

For this purpose, two panels made from Rogers RT/Duroid 6002 (0.635 mm thickness), collectively containing 246 PCBs, were processed using the optimal parameter settings derived from the predictive models and then submitted to a quality assurance check (Figure 6).

The dimensions specified in the client’s mechanical drawing were for a PCB measuring 36.81 mm × 16.62 mm, with a dimensional tolerance of ±0.1 mm for each geometrical feature.

For each PCB, both specified dimensions, the kerf angle, and the excess kerf depth were measured, and their means and standard deviations were calculated:

- Length: 36.81 mm ± 0.04 mm, measured along the longitudinal axis of the PCB.
- Height: 16.62 mm ± 0.03 mm, measured along the transverse axis.
- Kerf angle: 88.74° ± 0.32°, representing the inclination of the cut edge.
- Excess kerf depth: 40.95 µm ± 2.15 µm.

The results demonstrated a 100% success rate for geometric tolerances, surface integrity, and excess kerf depth prediction, confirming the reliability of the predictive models for industrial applications and again attesting to the robustness of the developed predictive models and the absence of overfitting.

This further validation confirms the models’ strong predictive capabilities across various PTFE-based substrates. Subsequent applications have also demonstrated the model’s effectiveness on several other materials, confirming its applicability to the Rogers 3000 series, Rogers RT/Duroid 5000 and 6000 series (Rogers Corporation, Chandler, Arizona, AZ, USA), and Taconic families (Taconic, Petersburgh, New York, NY, USA).

This model also enables the derivation of parameter sets capable of processing thicknesses ranging from about 0.17 mm to 1.10 mm.

## 4. Conclusions

This study successfully developed predictive mathematical models for CO_2_ laser kerf depth in PTFE-based PCB materials using a comprehensive Design of Experiments approach. A two-level full factorial design systematically investigated the influence of the number of loops, aperture, and pulse duration, revealing their individual and interactive effects on material removal. While the analysis identified pulse duration as exhibiting the most significant effect on the resultant ablation depth, the number of loops proved to be the most critical factor from a production operation perspective. This is due to its direct and often linear proportionality with kerf depth, facilitating precise control and mitigating heat accumulation in PTFE, which has inherently low thermal conductivity. Pulse duration, while impactful, can be operationally leveraged for optimizing process time by influencing the ablation depth per pulse. Conversely, the aperture, which defines the laser spot size and thus the energy density on the material, is often constrained by the geometric features and design specifications of the PCB itself.

The models developed for the RO3003 substrate demonstrated excellent generalizability when validated on Rogers RT/Duroid 5880 material, showing nearly identical significant factors and equation coefficients. This consistency validates the robustness of the developed predictive equations and the absence of overfitting, suggesting that the underlying ablation mechanisms are consistent across these PTFE-based laminates. Furthermore, validation of the production of PCBs made from Rogers RT/Duroid 6002 achieved a 100% success rate in meeting geometric tolerances and surface integrity requirements. This rigorous validation underscores the reliability and practical utility of the developed predictive framework for industrial applications. The proposed DoE-based approach establishes a robust methodological framework for defining process windows in CO_2_ laser micromachining of PTFE-based PCBs. While the specific mathematical coefficients are unique to the experimental setup used, the identified parametric trends and interaction effects provide a scalable roadmap for industrial routing operations on similar high-frequency substrates, enabling quantitative process control instead of empirical tuning. The derived predictive model significantly reduced parameter identification time and minimized scrap generation, thereby enhancing process control and improving manufacturing yield.

## Figures and Tables

**Table 1:** DoE matrix for RO3003 experimentation.

ID	A Aperture, μm	B Pulse Duration, μs	C Loops	Duty Cycle, %	Peak Power, MW/cm^2^	Energy per Pulse, mJ	Peak Fluence, J/cm^2^
−1	127	5	10	1.67	40.49	12.82	202.47
a	254	5	10	1.67	13.13	16.64	65.66
b	127	9	10	3.00	22.50	12.82	202.47
ab	254	9	10	3.00	7.30	16.64	65.66
c	127	5	20	1.67	40.49	12.82	202.47
ac	254	5	20	1.67	13.13	16.64	65.66
bc	127	9	20	3.00	22.50	12.82	202.47
abc	254	9	20	3.00	7.30	16.64	65.66

**Table 2:** Measurement results for RO3003 experimentation.

ID	$\mathrm{Mean}\;\mathrm{of}\;h_{p}$, μm
−1	225.7
a	171.8
b	552.6
ab	421.2
c	442.0
ac	342.4
bc	1082.0
abc	824.5
CP	225.7

**Table 3:** ANOVA results for the RO3003 experiment.

Source	Sum of Squares	DF	Mean Square	F-Value	*p*-Value	% of Contribution
Model	0.4996	3	0.1665	23,539.06	<0.0001	/
A, Aperture	0.0271	1	0.0271	3827.15	<0.0001	5.42
B, Pulse duration	0.2999	1	0.2999	42,390.46	<0.0001	60.02
C, Loops	0.1726	1	0.1726	24,399.57	<0.0001	34.55
Residual	0.0000	4	7.075 × 10^−6^	/	0.2679	0.01
Total	0.4996	7	0.3855	/	/	100.00
$R^{2}=0.9999{\;\;\;\;\;R}_{adj}^{2}=0.9999\;\;\;\;\;R_{pred}^{2}=0.9998\;\;\;\;\;RMSE=1.96\;\mu m$						

**Table 4:** DoE matrix for RT/Duroid 5880 experimentation.

ID	A Aperture, μm	B Pulse Duration, μs	C Loops	Duty Cycle, %	Peak Power, MW/cm^2^	Energy per Pulse, mJ	Peak Fluence, J/cm^2^
−1	127	5	10	1.67	40.49	12.82	202.47
a	254	5	10	1.67	13.13	16.64	65.66
b	127	9	10	3.00	22.50	12.82	202.47
ab	254	9	10	3.00	7.30	16.64	65.66
c	127	5	15	1.67	40.49	12.82	202.47
ac	254	5	15	1.67	13.13	16.64	65.66
bc	127	9	15	3.00	22.50	12.82	202.47
abc	254	9	15	3.00	7.30	16.64	65.66

**Table 5:** Measurement results for RT/Duroid 5880 experimentation.

ID	$\mathrm{Mean}\;\mathrm{of}\;h_{p}$, μm
−1	225.2
a	171.3
b	551.1
ab	422.7
c	331.1
ac	255.3
bc	807.3
abc	625.8

**Table 6:** ANOVA results for RT/Duroid 5880 experiment.

Source	Sum of Squares	DF	Mean Square	F-Value	*p*-Value	% of Contribution
Model	0.3866	3	0.1289	24,428.75	<0.0001	/
A, Aperture	0.0262	1	0.0262	4961.49	<0.0001	6.77
B, Pulse duration	0.3032	1	0.3032	57,469.70	<0.0001	78.41
C, Loops	0.0573	1	0.0573	10,855.06	<0.0001	14.81
Residual	0.0000	4	5.275 × 10^−6^	/	/	0.01
Total	0.3866	7	0.3855	/	/	100.00
$R^{2}=0.9999{\;\;\;\;\;R}_{adj}^{2}=0.9999\;\;\;\;\;R_{pred}^{2}=0.9998\;\;\;\;\;RMSE=1.89\;\mu m$						

## Data Availability

The original contributions presented in this study are included in the article/Supplementary Materials. Further inquiries can be directed to the corresponding author.

## Supplementary Materials

The following supporting information can be downloaded at: https://www.mdpi.com/article/10.3390/mi17040404/s1. Figure S1. Box-Cox plot for RO3003 model. Figure S2. Normal probability plot for RO3003 model. Figure S3. Residuals vs. predicted plot for RO3003 model. Figure S4. Residuals vs. run plot for RO3003 model. Figure S5. Cook’s distance plot for RO3003 model. Figure S6. Normal probability plot for RT/Duroid 5880 model. Figure S7. Residuals vs. predicted plot for RT/Duroid 5880 model. Figure S8. Box-Cox plot for RT/Duroid 5880 model. Figure S9. Residuals vs. run plot for RT/Duroid 5880 model. Figure S10. Cook’s distance plot for RT/Duroid 5880 model.
