# Supplementary material for: CO2 Laser Micromachining of PTFE-Based PCBs: Predictive Modeling of Kerf Depth Through Design of Experiments
Source: Micromachines (Basel). 2026 Mar 26;17(4):404. doi: 10.3390/mi17040404 (PMC13117439; doi:10.3390/mi17040404)
Supplement: Supplementary file 1 [file micromachines-17-00404-s001.zip › micromachines-4221939-supplementary.pdf]

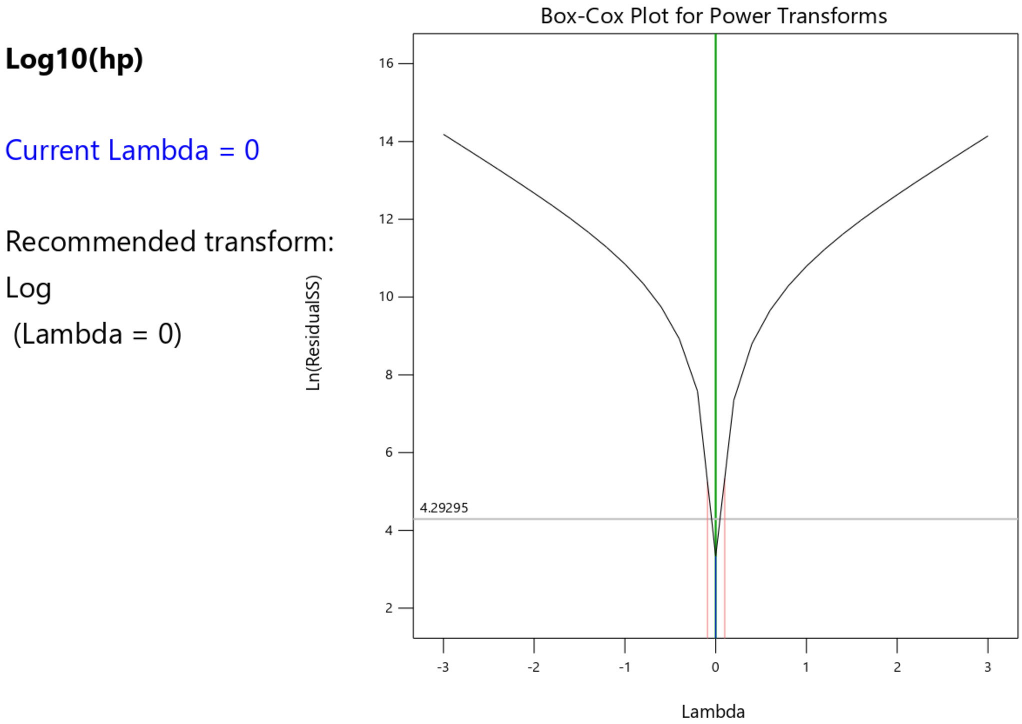

Figure S1. Box-Cox plot for RO3003 model

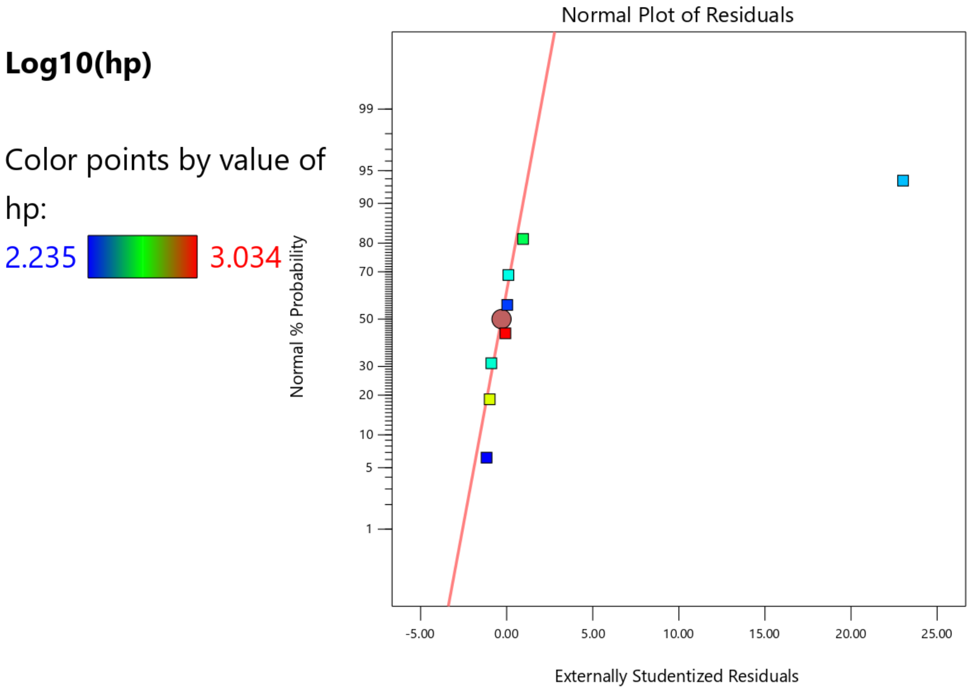

Figure S2. Normal probability plot for RO3003 model

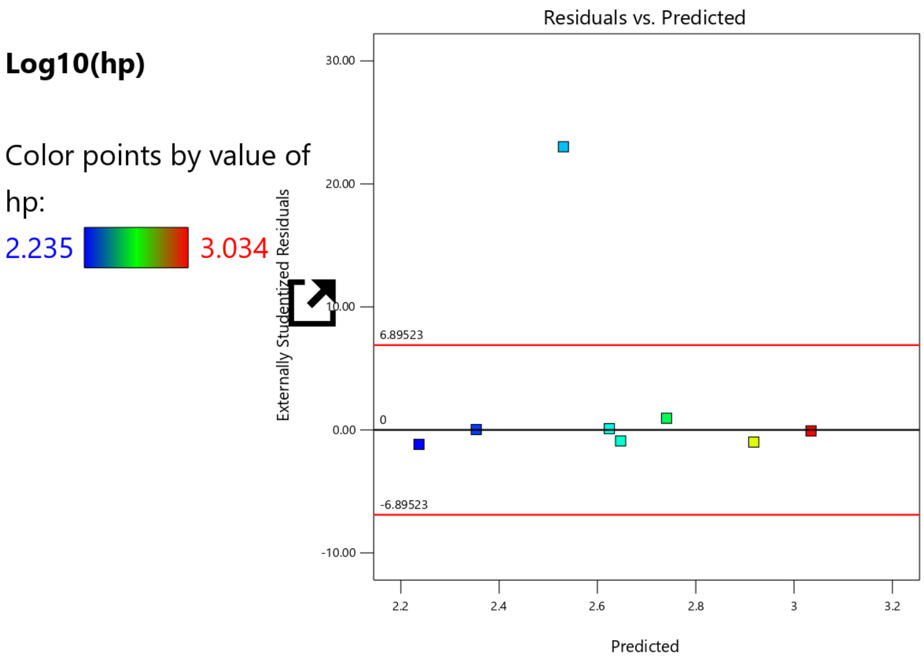

Figure S3. Residuals vs predicted plot for RO3003 model

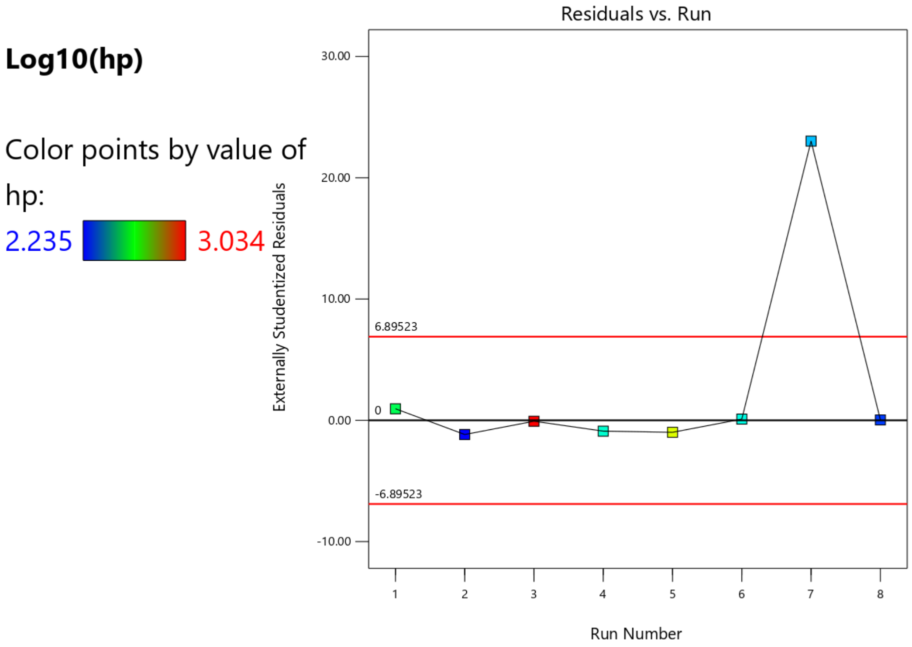

Figure S4. Residuals vs run plot for RO3003 model

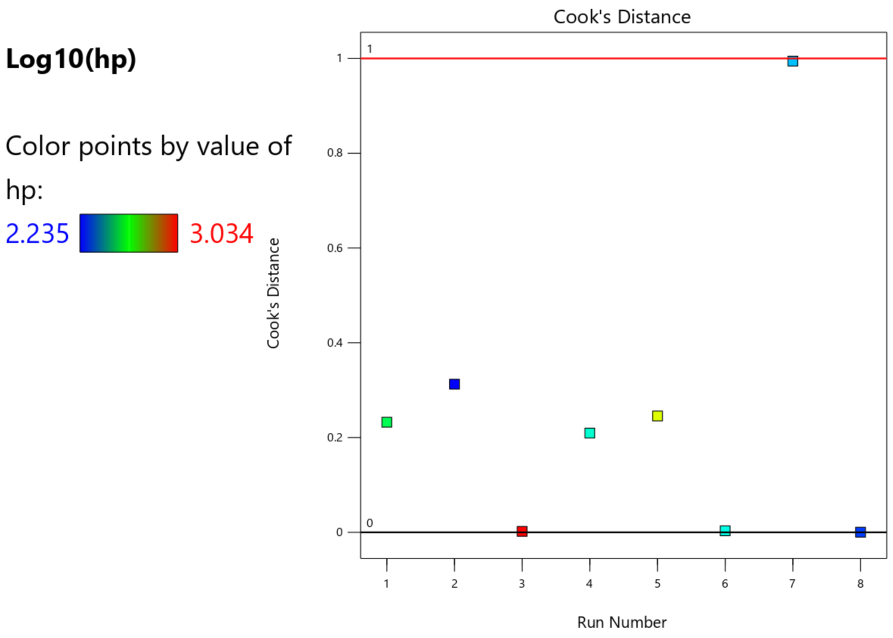

Figure S5. Cook's distance plot for RO3003 model

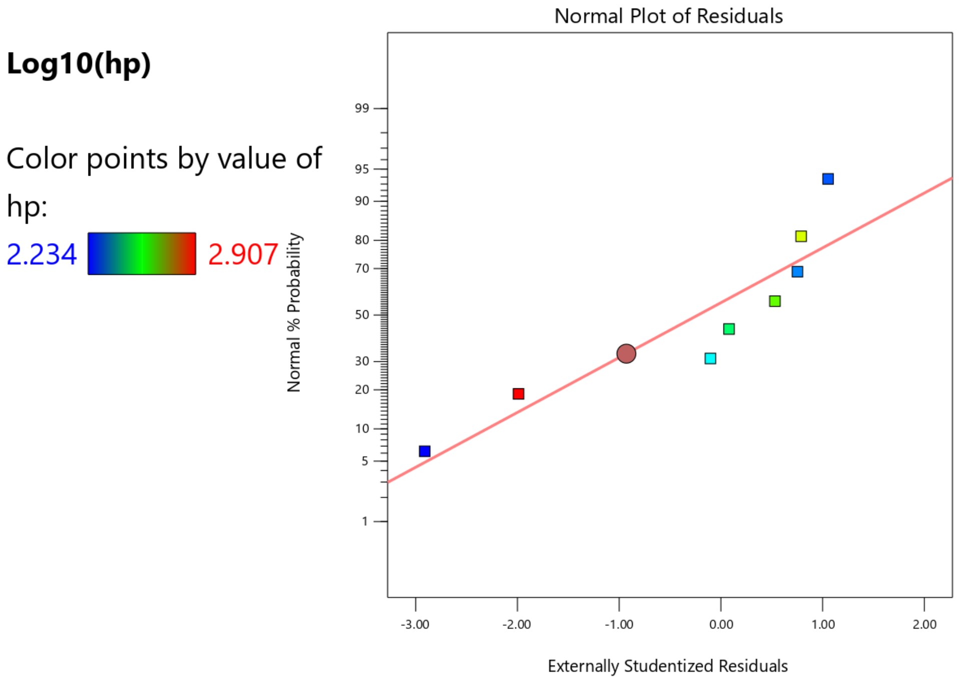

Figure S6. Normal probability plot for RT/Duroid 5880 model

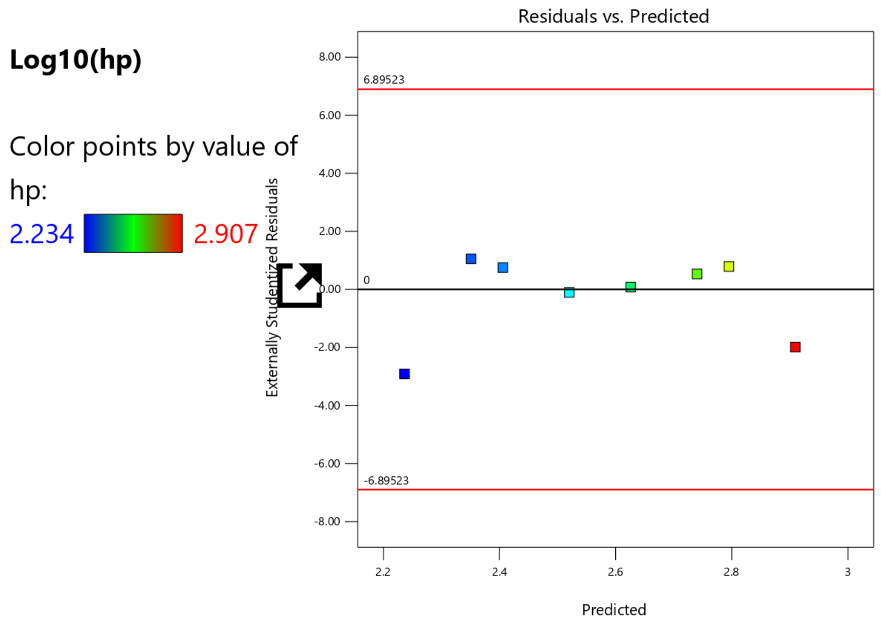

Figure S7. Residuals vs predicted plot for RT/Duroid 5880 model

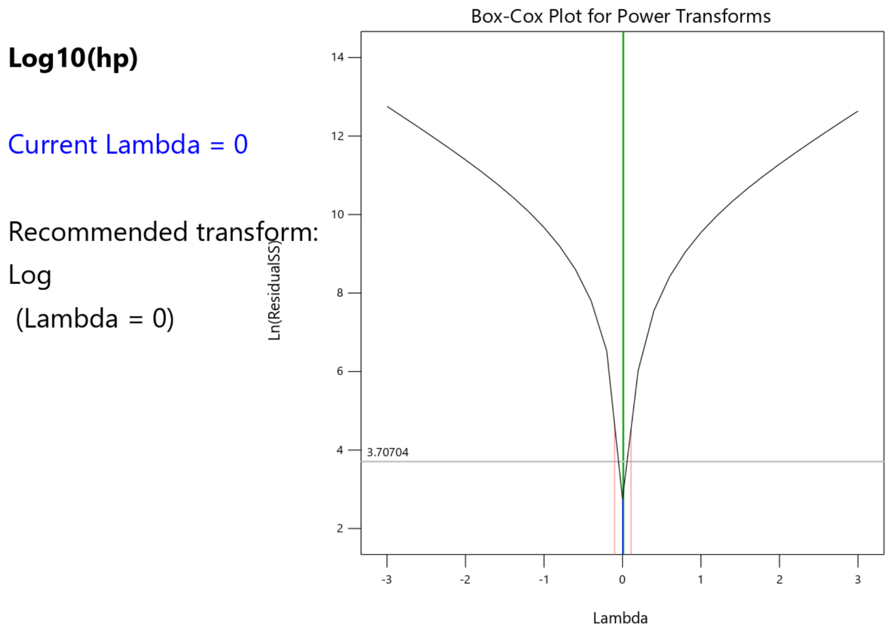

Figure S8. Box-Cox plot for RT/Duroid 5880 model

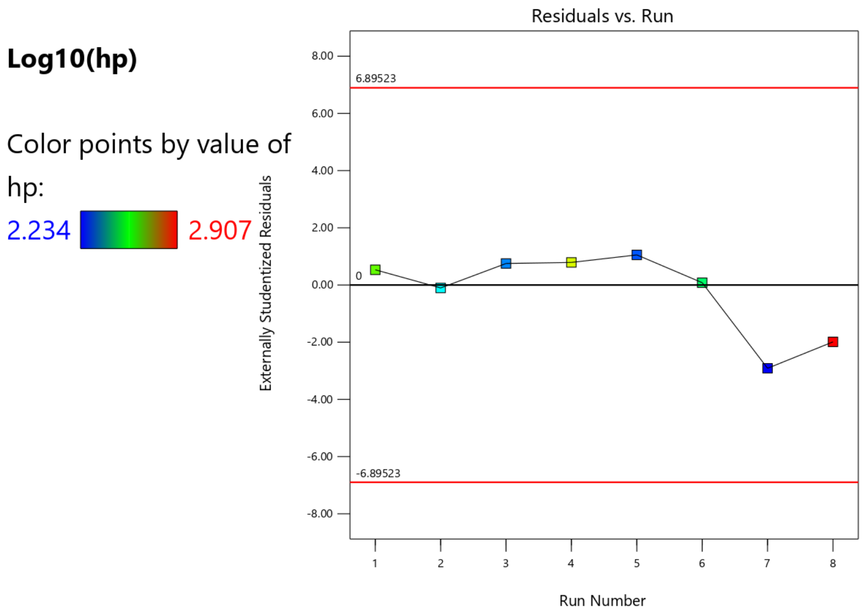

Figure S9. Residuals vs run plot for RT/Duroid 5880 model

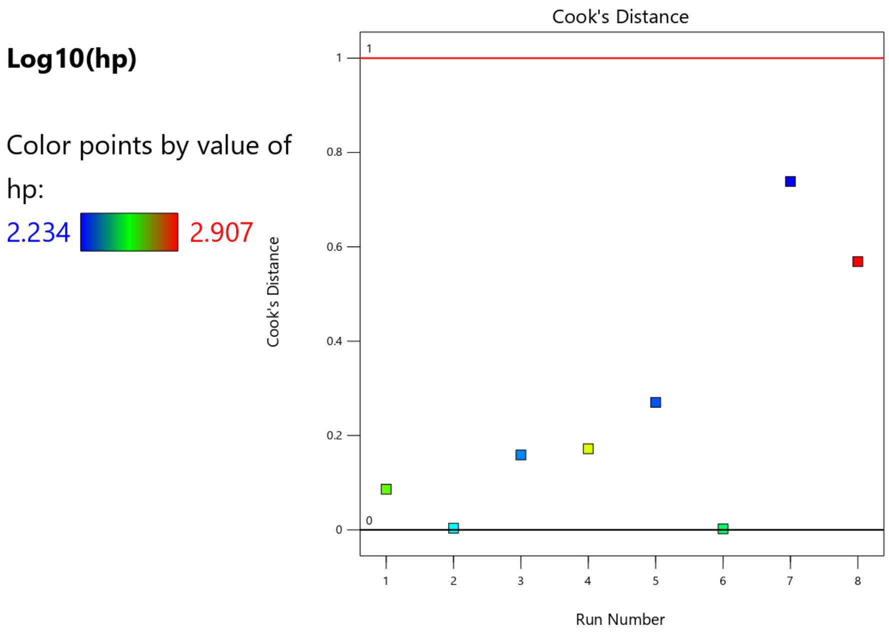

Figure S10. Cook’s distance plot for RT/Duroid 5880 model
